# Appropriateness of referrals to Whiston hospital Mental Health Liaison Services

**DOI:** 10.1192/bjo.2021.931

**Published:** 2021-06-18

**Authors:** Nick Strouther, Divya Jain

**Affiliations:** The Brooker Centre, HENW

## Abstract

**Aims:**

1. The aim of this study was to assess the appropriateness of referrals to Whiston Mental Health Liaison Services (WMHLS) according to Royal College of Psychiatrists and local trust guidelines.

2. To assess whether the referrals were being reviewed in timely manner as per the trust's guidelines.

**Method:**

Data collection was completed using a proforma to ensure uniform data collection. The proforma included information on patient demographics, previous mental health service involvement, other details like reasons and time of referral and their outcomes. Data sample comprised of 46 patients who had been referred to the WMHLS in the month of August 2019 were randomly selected.

**Result:**

44 of the 46 referrals analyzed were found to be appropriate. 40 patients were deemed to have appropriate documentation. The ratio of males to females was 20:26. 21 referrals were from the observation ward, 14 from A&E, and 11 from medical wards. 40 patients were previously known to mental health services. The reasons for referral ranged from suicidal ideation/attempt (48%), Drug related (12%), Assessment (7%) and more. There were various outcomes recorded. One of them was that 18 (28%) referrals were assessed for Depression and for other mental health problems.

78.6% of patients referred from A&E, and 95.2% of patients in the observation ward, were not seen in the 1 hour window set out by the Trust's guidelines. 91.1% of patients referred from the wards were seen within the 24 hour target.

**Conclusion:**

The vast majority of referrals were found to be appropriate (44/46). It was found that the referral form used across the Trust, contained different levels of details and information on the patient depending on the source of referral. Using a standard process to complete referral forms to be used across the whole trust may ensure that all patients receive a standardized and appropriate referral based on the guidelines. Making the form electronic may reduce problems deciphering handwriting, and could allow WMHLS have a better understanding of the patient, and allow them to identify a patient that may be more appropriate for another service, e.g. drugs and alcohol team. This may and make the overall referral process quicker and reduce waiting times in A&E, as well as faster referrals to the appropriate services.

